# The combination of arsenic, interferon-alpha, and zidovudine restores an “immunocompetent-like” cytokine expression profile in patients with adult T-cell leukemia lymphoma

**DOI:** 10.1186/1742-4690-10-91

**Published:** 2013-08-20

**Authors:** Ghada Kchour, SA Rahim Rezaee, Reza Farid, Akram Ghantous, Houshang Rafatpanah, Mahdi Tarhini, Mohamad-Mehdi Kooshyar, Hiba El Hajj, Fadwa Berry, Mohamad Mortada, Roudaina Nasser, Abbas Shirdel, Zeina Dassouki, Mohamad Ezzedine, Hossein Rahimi, Ardeshir Ghavamzadeh, Hugues de Thé, Olivier Hermine, Mahmoud Mahmoudi, Ali Bazarbachi

**Affiliations:** 1 Department of Biology, Faculty of Sciences, Lebanese University, Hadath, Lebanon; 2 Microbiology and Virology Research Center, Bu-Ali Research institute, Mashhad University of Medical Sciences, Mashhad, Iran; 3 Immunology Research Centre Bu-Ali Research Institute, Mashhad University of Medical Sciences, Mashhad, Iran; 4 Lebanese American University, School of Arts and Sciences, Beirut, Lebanon; 5 Faculty of Nursing Sciences, Islamic University, Beirut, Lebanon; 6 Department of Internal Medicine, Mashhad University of Medical Sciences, Mashhad, Iran; 7 Department of Internal Medicine, American University of Beirut, Beirut, Lebanon; 8 Tehran University of Medical Sciences, Tehran, Iran; 9 INSERM UMR 944 and CNRS UMR 7212, Hôpital Saint Louis, Paris, France; 10 CNRS UMR 8147, Hôpital Necker, Paris, France

**Keywords:** Arsenic, Interferon, Zidovudine, HTLV-I, ATL, Cytokines, Immune deficiency

## Abstract

**Background:**

HTLV-I associated adult T-cell leukemia/lymphoma (ATL) carries a dismal prognosis due to chemo-resistance and immuno-compromised micro-environment. The combination of zidovudine and interferon-alpha (IFN) significantly improved survival in ATL. Promising results were reported by adding arsenic trioxide to zidovudine and IFN.

**Results:**

Here we assessed Th1/Th2/T_reg_ cytokine gene expression profiles in 16 ATL patients before and 30 days after treatment with arsenic/IFN/zidovudine, in comparison with HTLV-I healthy carriers and sero-negative blood donors. ATL patients at diagnosis displayed a T_reg_/Th2 cytokine profile with significantly elevated transcript levels of Foxp3, interleukin-10 (IL-10), and IL-4 and had a reduced Th1 profile evidenced by decreased transcript levels of interferon-γ (IFN-γ) and IL-2. Most patients (15/16) responded, with CD4^+^CD25^+^ cells significantly decreasing after therapy, paralleled by decreases in Foxp3 transcript. Importantly, arsenic/IFN/zidovudine therapy sharply diminished IL-10 transcript and serum levels concomittant with decrease in IL-4 and increases in IFN-γ and IL-2 mRNA, whether or not values were adjusted to the percentage of CD4^+^CD25^+^ cells. Finally, IL-10 transcript level negatively correlated with clinical response at Day 30.

**Conclusions:**

The observed shift from a T_reg_/Th2 phenotype before treatment toward a Th1 phenotype after treatment with arsenic/IFN/zidovudine may play an important role in restoring an immuno-competent micro-environment, which enhances the eradication of ATL cells and the prevention of opportunistic infections.

## Background

Adult T-cell leukemia/lymphoma (ATL) is an aggressive malignancy of mature activated T-cells caused by human T cell lymphotropic virus type I (HTLV-I) [1,2]. ATL carries a very bad prognosis because of intrinsic chemo-resistance [3-6]. Moreover, ATL patients are functionally severely immunocompromised and may develop a variety of opportunistic infections, which further contribute to the poor prognosis [7-9]. These include cytomegalovirus, *Pneumocystis carinii* pneumonias, malignant strongyloidosis, crusted (Norwegian) scabies, disseminated cryptococcosis, toxoplasmosis, or fungal infections, as well as bacterial infections particulary of the respiratory tract or of the lower urinary, and septicemias.

HTLV-1 primarily infects CD4^+^ T helper (Th) cells that play a central role in adaptive immune responses. These Th cells are the predominant viral reservoir in the peripheral blood [10] and are normally classified into four major lineages: Th1, Th2, Th17, and T regulatory (T_reg_) cells, which produce interferon-γ (IFN-γ), interkeukin-4 (IL-4), IL-17, and IL-10, respectively [11]. T_reg_ are a subset of CD4^+^CD25^+^ T cells characterized by the expression of the transcriptional regulator Foxp3 [12] and the secretion of high levels of IL-10. In healthy individuals, T_reg_ cells maintain immune homeostasis and protect against effector responses to autoantigens or over-exuberant responses to exogenous antigens [13]. Th1 effector cells produce IFN-γ and IL-2, play a critical role in cellular immunity against viral infections, and determine a greater inflammatory response. Th2 cells produce IL-4 and stimulate humoral or allergic responses. Under normal conditions, there is a Th1/Th2 cytokine balance that is disrupted when infectious agents induce an overproduction of the Th2 cytokines, leading to the inhibition of the adaptive immune response against the pathogen.

The immune-suppression in ATL patients is likely mediated by cytokines directly produced by the ATL cells or by ATL-triggered disruption of the normal cytokine balance produced by normal immune cells. Furthermore, ATL cells may directly function as T_reg_ cells and suppress normal effector T cells [14-17]. Overall, this immunosuppressive micro-environment enables ATL cells to evade the host immune response. Unfortunately, chemotherapy further exacerbate this phenomenon.

After 30 years of research on HTLV-I and associated diseases, treatment of ATL patients remains a challenge [3,6,18-20]. In acute ATL, Japanese trials demonstrated that although chemotherapy combinations improve response rate, they fail to achieve a significant impact on survival [21,22]. Patients with chronic and smoldering ATL have a better prognosis but long-term survival is poor when these patients are managed with a watchful-waiting policy or with chemotherapy [23]. Recently, a worldwide meta-analysis revealed that the combination of zidovudine and interferon-alpha (IFN) is highly effective in the leukemic subtypes of ATL and should be considered as standard first line therapy in that setting [24]. This combination has changed the natural history of the disease through achievement of significantly improved long-term survival in patients with smoldering and chronic ATL as well as a subset of patients with acute ATL [24]. ATL lymphoma patients still benefit from chemotherapy induction with concurrent or sequential antiretroviral therapy with zidovudine and IFN. Yet, most patients relapse and alternative therapies are mandatory. In prior studies, using an *in vitro* model of ATL derived cell lines and freshly isolated ATL leukemic cells, we showed that arsenic trioxide synergizes with IFN to induce G1 arrest and apoptosis in ATL [25] through shut-off of the NF-кB pathway and Tax degradation by the proteasome [26,27]. This combination yielded promising clinical results in relapsed/refractory ATL patients [28]. We recently showed that arsenic/IFN combination cures ATL mice through selective targeting of leukemia initiating cell (LIC) activity [29]. Finally, we reported an unprecedented 100% response rate including 70% complete remission rate in newly diagnosed chronic ATL patients treated with the combination of arsenic, interferon and zidovudine (arsenic/IFN/zidovudine) [30].

In the current study, we investigated the effect of the triple combination of arsenic/IFN/zidovudine on the immune micro-environment in ATL patients. We show that ATL patients at diagnosis displayed a T_reg_/Th2 cytokine production profile. Strikingly, after therapy, the cytokine production balance shifted from this initial “immunosuppressive-like” state towards an “immunocompetent-like” state (Th1 profile). This study provides insights on the mechanism of action of this potentially curative combination on the immune micro-environment in ATL patients. This immunological switch may participate in the defense against opportunistic infections as well as in the anti-tumor immunity.

## Results

### Arsenic/IFN/zidovudine treatment induced a high response rate in ATL patients

Sixteen previously untreated ATL patients (2 acute ATL, 2 ATL lymphoma, and 12 chronic ATL) received arsenic/IFN/zidovudine treatment. The patients’ characteristics are listed in Table 1. Clinical data, response to therapy and follow up data were previously reported for 10 of these patients [30]. Briefly, all patients initially presented with symptomatic disease requiring treatment. The most frequent symptoms were cutaneous manifestations with maculopapular rash, severe itching, and skin ulcerations. At day 30 and as previously reported [30], treatment with arsenic, IFN, and AZT resulted in a good albeit partial response in all patients except one ATL lymphoma: 9 patients achieved partial response (PR) and 6 patients achieved very good partial response (VGPR), as described in Patients and Methods. One ATL lymphoma died from disease progression (Table 1). Interestingly, as previously reported in seven patients [30], in the eleven patients for whom initial and Day 30 DNA was available, HTLV-I proviral load significantly decreased from an average of 1415 copy/μl of blood to 226 copy/μl (p<0.05). Most patients continued to improve their response and the best response was achieved within 2 to 4 months. Indeed, out of the 15 responding patients, 10 patients achieved CR, 2 patients achieved VGPR (solely because of the presence of 6% and 8% of atypical lymphocytes on peripheral blood smear, respectively), and three patients achieved PR (Table 1).

**Table 1:** Patients characteristics

**Patient age**	**Gender**	**ATL subtype**	**Response at day 30**	**Best response**	**Initial viral load (copy/μl)**	**Viral load at day 30 (copy/μl)**
58	F	Acute	PR	PR	105483	219
56	M	Acute	PR	PR	NA	NA
60	M	Chronic	PR	CR	NA	NA
47	F	Chronic	VGPR	CR	1990	336
53	M	Chronic	PR	CR	84	33
72	M	Chronic	VGPR	CR	548	0
36	M	Chronic	VGPR	VGPR	999	838
46	F	Chronic	VGPR	CR	NA	NA
63	F	Chronic	PR	CR	1081	63
51	F	Chronic	PR	CR	196	64
68	F	Chronic	VGPR	VGPR	1805	65
60	M	Chronic	PR	CR	47	7
53	M	Chronic	PR	PR	49724	18898
77	M	Chronic	VGPR	CR	3747	182
54	M	Lymphoma	NR	NR	NA	NA
48	M	Lymphoma	PR	CR	NA	NA

CR= complete remission; PR= partial response; VGPR= very good partial response; NR= No response; NA= not available.

We examined the effect of treatment on the relative distribution of the T cell subpopulations at day 30 (Table 2). Treatment with arsenic, AZT, and IFN decreased the average percentage of CD4^+^ cells (from 87 ± 14% to 77 ± 21%; p<0.01) and increased the average percentages of CD8^+^ cells (from 12 ± 12% to 16 ± 12%; p<0.01). In accordance with the observed response to therapy, a decrease in the average percentage of CD4^+^CD25^+^ cells was noted in all patients after treatment (from 47 ± 15% to 25 ± 14%; p<0.001), suggesting that this combination is mainly acting on the circulating malignant cells and HTLV-I infected cells (Table 2).

**Table 2:** Flow cytometry analysis of T cell surface markers at initiation (Before) and 30 days after (After) treatment with arsenic/IFN/zidovudine, SD, standard deviation

**Patient age**	**CD4+ cell/μl**		**% CD4+ cells**		**CD8+ cell/μl**		**% CD8+ cells**		**%CD4+/ CD25+ cells**	
	**Before**	**After**	**Before**	**After**	**Before**	**After**	**Before**	**After**	**Before**	**After**
58	185710	90528	100	96	1857	1886	1	2	52	32
56	77040	2592	100	89	770	262	1	9	65	41
60	7020	3240	82	82	1320	1120	14	18	53	35
47	250	194	90	98	6318	3175	8	12	55	33
53	779	594	59	53	488	459	37	41	50	14
72	31142	4317	91	86	3080	703	9	14	44	25
36	5100	1584	93	90	5050	1095	7	10	48	42
46	4989	2072	85	67	3934	2360	15	33	58	22
63	4335	1061	80	42	765	523	20	28	41	7
51	1010	307	98	94	4269	1650	2	6	18	10
68	4989	1989	100	96	50	21	1	1	50	15
60	48370	15657	82	82	7980	2730	14	18	25	18
53	274	84	93	89	44984	13935	7	11	70	54
77	4469	1119	56	41	2873	764	36	28	52	25
54	1333	304	80	49	300	112	18	18	16	7
48	2160	2226	100	96	1	1	1	1	55	26
**Mean**			**87**	**77**			**12**	**16**	**47**	**25**
**SD**			**14**	**22**			**12**	**12**	**15**	**14**
**P value**			**<0.01**				**<0.01**		**<0.001**	

P values indicate the significance level for the pairwise comparisons between “Before” and “after”.

### Arsenic/IFN/zidovudine treatment decreased transcript levels of Treg and Th2 markers

We investigated the early effect of the triple combination (arsenic/IFN/zidovudine) on the T_reg_ subpopulation by evaluating the transcript levels of Foxp3 and IL-10 in 10 healthy sero-negative blood donors, 10 asymptomatic HTLV-I healthy carriers, as well as in 16 ATL patients before and 30 days after treatment, before achievement of maximal response.

**Figure 1:**
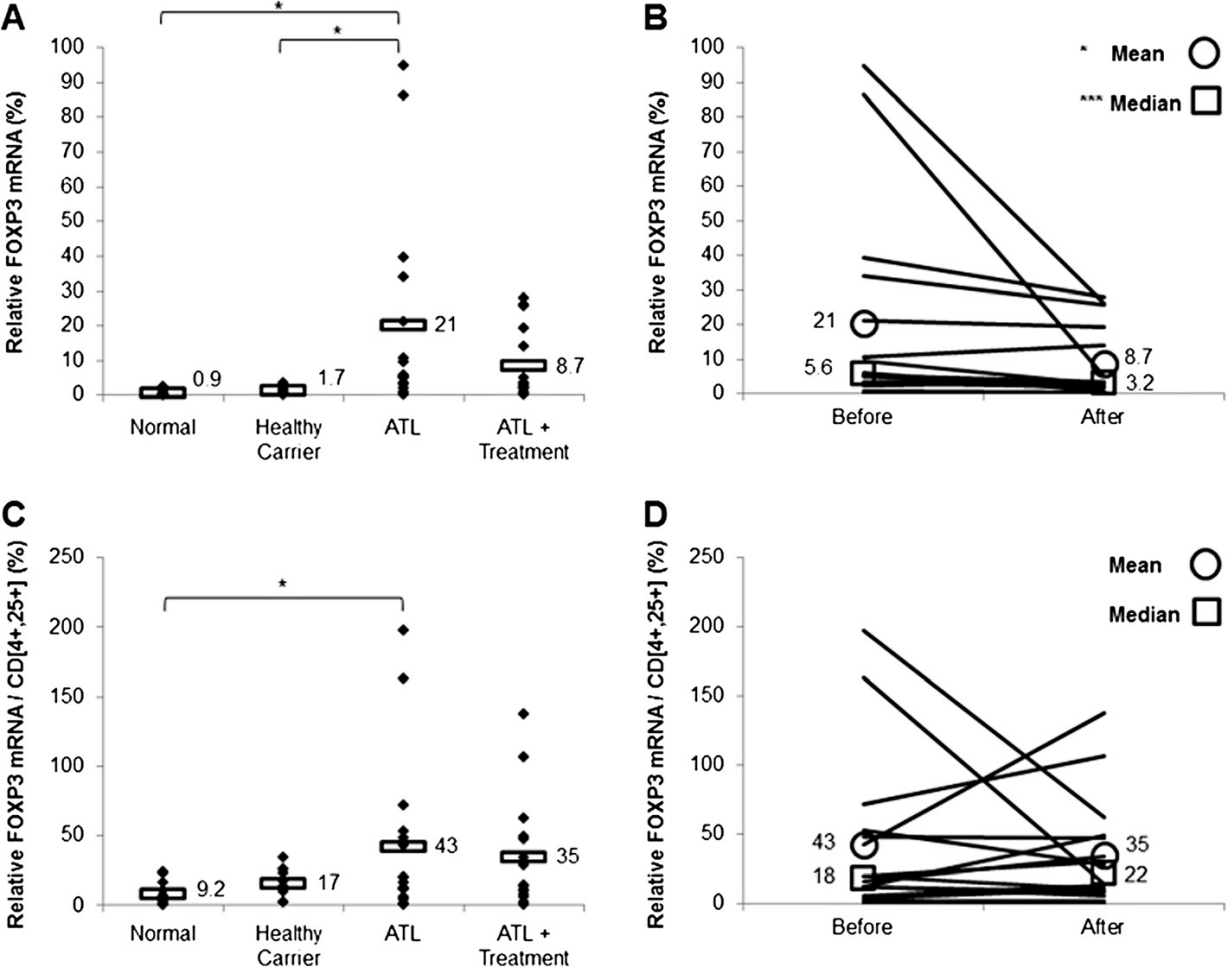
**Treatment with arsenic/IFN/zidovudine decreased Foxp3 expression. A**. Foxp3 transcript levels in normal blood donors (n=10), healthy carriers of HTLV-I (n=10) and ATL patients (n=16) at initiation and 30 days after treatment with arsenic/IFN/zidovudine. Rectangles represent mean values. **B**. Mean and median Foxp3 transcript levels of individual ATL patients at initiation and 30 days after treatment with arsenic/IFN/zidovudine. **C**, **D**. Relative expression of Foxp3 transcripts after normalization to the number of CD4^+^CD25^+^ ATL cells. All values are expressed as percentage of human beta2-microglobulin used as internal control. *, **, *** indicate p values less than 0.05, 0.01 and 0.001 respectively.

**Figure 2:**
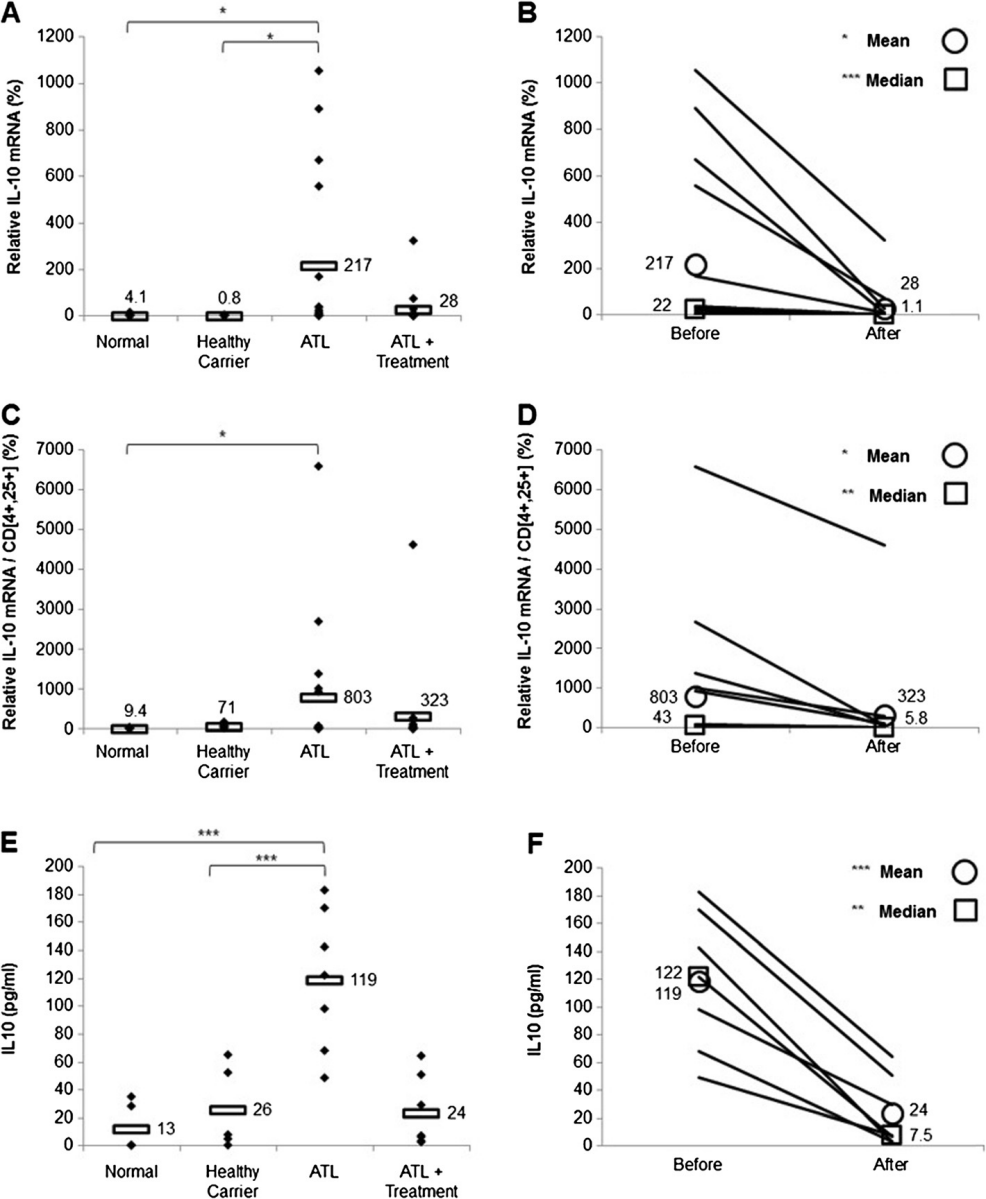
**Treatment with arsenic/IFN/zidovudine decreased IL-10 expression and serum levels. A**. IL-10 transcript levels in normal blood donors (n=10), healthy carriers of HTLV-I (n=10) and ATL patients (n=16) at initiation and 30 days after treatment with arsenic/IFN/zidovudine. Rectangles represent mean values. **B**. Mean and median IL-10 transcript levels of individual ATL patients at initiation and 30 days after treatment with arsenic/IFN/zidovudine. **C**, **D**. Relative expression of IL-10 transcripts after normalization to the number of CD4^+^CD25^+^ ATL cells. All values are expressed as percentage of human beta2-microglobulin used as internal control. **E**, **F**. Levels of secreted IL-10 in the serum of the seven ATL patients who displayed elevated pretreatment IL-10 serum level, measured by ELISA before and after treatment. *, **, *** indicate p values less than 0.05, 0.01 and 0.001 respectively.

Untreated ATL patients displayed significantly higher transcript levels of Foxp3 (Figure 1A) and IL-10 (Figure 2A), relative to seronegative or healthy carrier individuals (p < 0.05). Notably, Foxp3 and IL-10 mRNA levels in untreated ATL patients were, respectively, at least 12 and 53 folds higher than seronegative and healthy carrier individuals (p < 0.05). High interindividual variation was observed among ATL patients for both Foxp3 and IL-10 transcript levels (Figures 1A and 2A, Table 3). Interestingly, treatment with arsenic/IFN/zidovudine significantly decreased transcript levels of both Foxp3 (Figure 1B) and IL-10 (Figure 2B). Because ATL cells express a T_reg_ phenotype, we normalized the expression levels of Foxp3 and IL-10 relative to the percentage of CD4^+^ CD25^+^ cells. ATL patients still showed significantly higher normalized transcript levels of Foxp3 and IL-10 relative to seronegative individuals (Figures 1C and 2C; p < 0.05). Normalized mRNA values of IL-10 (Figure 2D), but not Foxp3 (Figure 1D), significantly decreased in ATL patients after treatment (p < 0.05). Interestingly, standardized IL-10 transcript level negatively correlates with clinical response at Day 30 (r = −0.452, p < 0.05, spearman). This correlation remained valid even when adjusted for the confounder effects of gender and/or age. This suggests that the lower the IL-10 levels before therapy, the better the response after 30 days of therapy.

**Table 3:** Foxp3 and cytokine transcript levels at initiation (Before) and 30 days after (After) treatment with arsenic/IFN/zidovudine

**Patient age**	**Foxp3 before**	**Foxp3 after**	**IL-10 before**	**IL-10 after**	**IL-4 before**	**IL-4 after**	**INF-γ before**	**INF-γ after**	**IL-2 before**	**IL-2 after**
58	0	0	0	0	0	0	13	80	7	34
56	16	34	1372	66	2	0	4	28	9	22
60	163	15	70	6	0	0	4	16	1	6
47	4	11	69	4	3	4	5.9	18	17	64
53	43	138	34	4	12	10	11.8	124	8	67
72	12	8	0	0	2	0	0.3	16	0	0
36	197	62	2	0	7	1	4.3	17	3	32
46	6	13	2	3	0	0	0.8	8	3	43
63	12	50	14	11	3	3	7	57	1	12
51	53	28	927	112	19	3	5	22	1	3
68	12	6	23	6	1	1	6	62	1	4
60	20	11	2684	60	8	4	3	155	1	4
53	48	47	0	0	0	0	2	7	0	0
77	2	2	52	6	3	3	13	45	1	4
54	20	30	6584	4602	5	1	5	114	0	2
48	72	107	1011	286	3	2	6	25	10	45

All values are expressed as percentage of human beta2-microglobulin used as internal control.

In order to confirm the decrease of IL-10 in treated patients, we assessed the secretion of this cytokine in the serum of patients by ELISA before and 30 days after treatment. Seven patients had significantly higher IL-10 serum levels relative to seronegative and healthy carrier individuals (Figure 2E; p < 0.001) and all displayed decreases in IL-10 serum levels after treatment (Figure 2F; p < 0.01). Interestingly, these seven patients are those who displayed the highest IL-10 mRNA expression before treatment (Figure 2A). IL-10 serum levels were low before and after treatment in the remaining patients (data not shown). These results suggest that treatment with arsenic/IFN/zidovudine decreases the proportion of ATL cells with T_reg_ phenotype as well as their IL-10 production.

**Figure 3:**
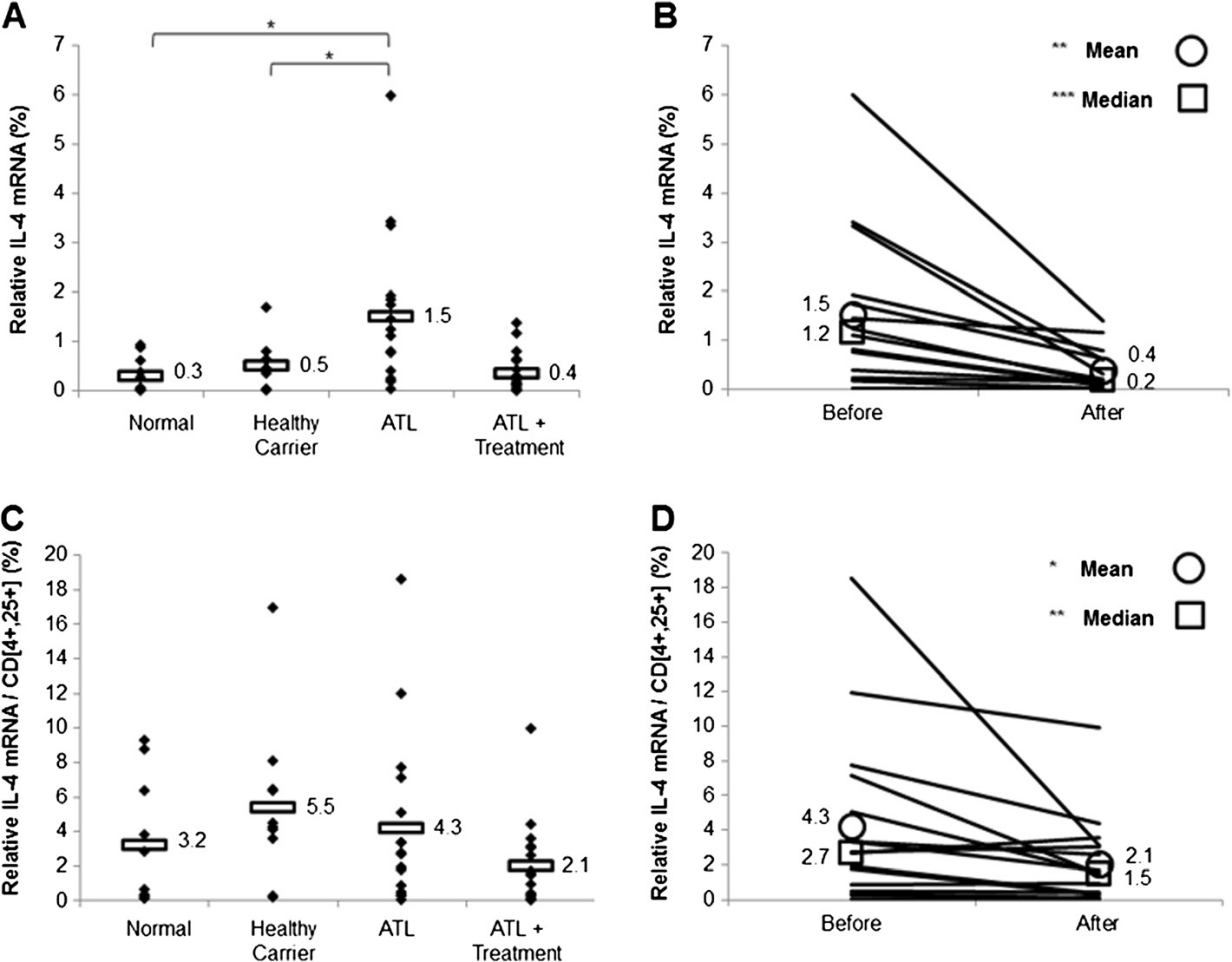
**Treatment with arsenic/IFN/zidovudine decreased IL-4 expression. A**. IL-4 transcript levels in normal blood donors (n=10), healthy carriers of HTLV-I (n=10) and ATL patients (n=16) at initiation and 30 days after treatment with arsenic/IFN/zidovudine. Rectangles represent mean values. **B**. Mean and median IL-4 transcript levels of individual ATL patients at initiation and 30 days after treatment with arsenic/IFN/zidovudine. **C**, **D**. Relative expression of IL-4 transcripts after normalization to the number of CD4^+^CD25^+^ ATL cells. All values are expressed as percentage of human beta2-microglobulin used as internal control. *, **, *** indicate p values less than 0.05, 0.01 and 0.001 respectively.

We then assessed the effect of treatment on the Th2 subpopulation by evaluating the transcript levels of IL-4. ATL patients had significantly higher levels of IL-4 mRNA (Figure 3A; p < 0.05), but not of normalized IL-4 transcripts (Figure 3C), compared to healthy carriers and seronegative blood donors. Treatment with arsenic/IFN/zidovudine significantly decreased IL-4 and normalized IL-4 expression (Table 3 and Figure 3B and 3D; p < 0.05). Interestingly, the decrease in IL-4 expression was more pronounced in the patients who had the highest pretreatment transcript levels (Table 3 and Figure 3B and D).

### Treatment with arsenic/IFN/zidovudine increased transcript levels of Th1 markers

**Figure 4:**
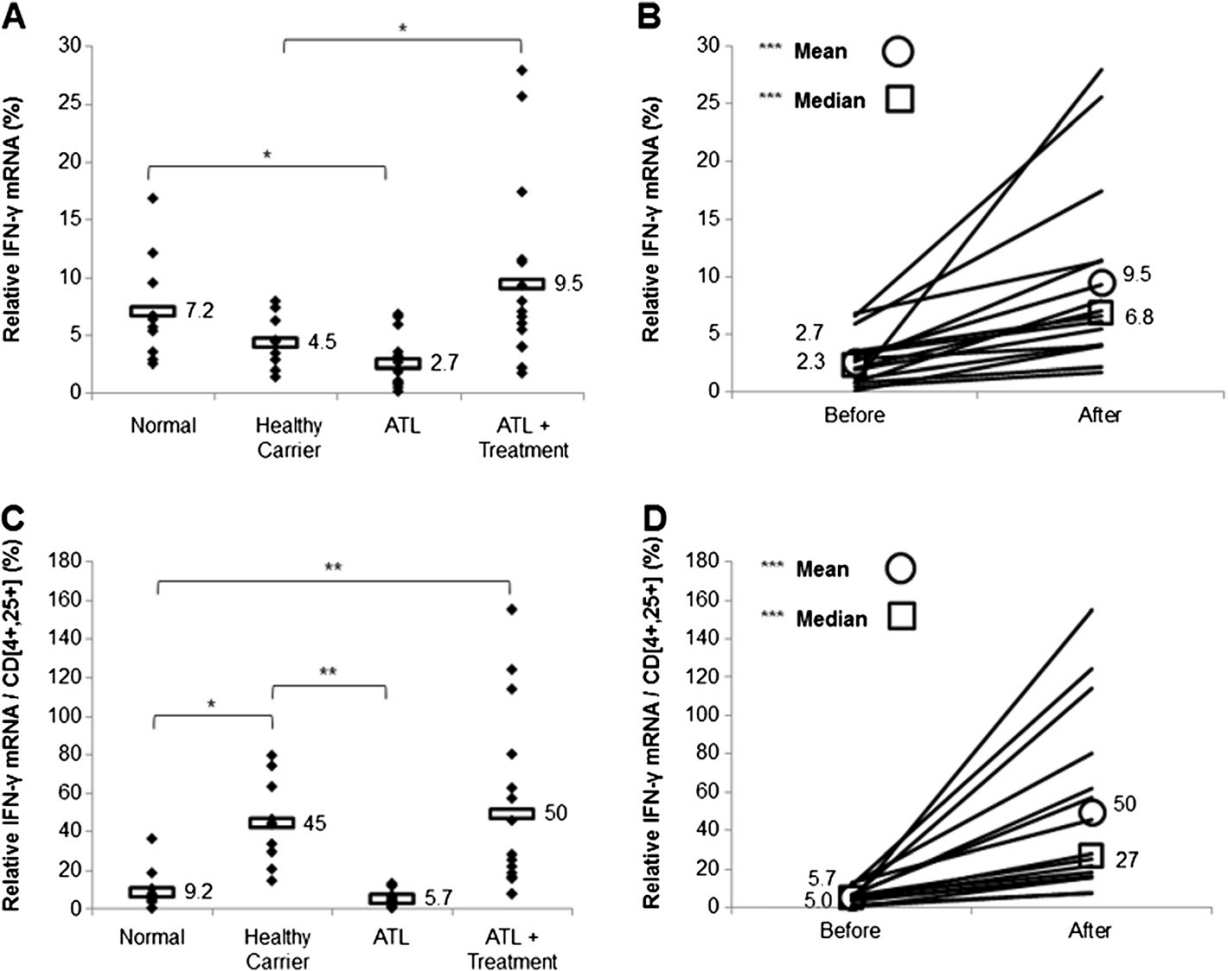
**Treatment with arsenic/IFN/zidovudine increased IFN-γ expression. A**. IFN-γ transcript levels in normal blood donors (n=10), healthy carriers of HTLV-I (n=10) and ATL patients (n=16) at initiation and 30 days after treatment with arsenic/IFN/zidovudine. Rectangles represent mean values. **B**. Mean and median IFN-γ transcript levels of individual ATL patients at initiation and 30 days after treatment with arsenic/IFN/zidovudine. **C**, **D**. Relative expression of IFN-γ transcripts after normalization to the number of CD4^+^CD25^+^ ATL cells. All values are expressed as percentage of human beta2-microglobulin used as internal control. *, **, *** indicate p values less than 0.05, 0.01 and 0.001 respectively.

**Figure 5:**
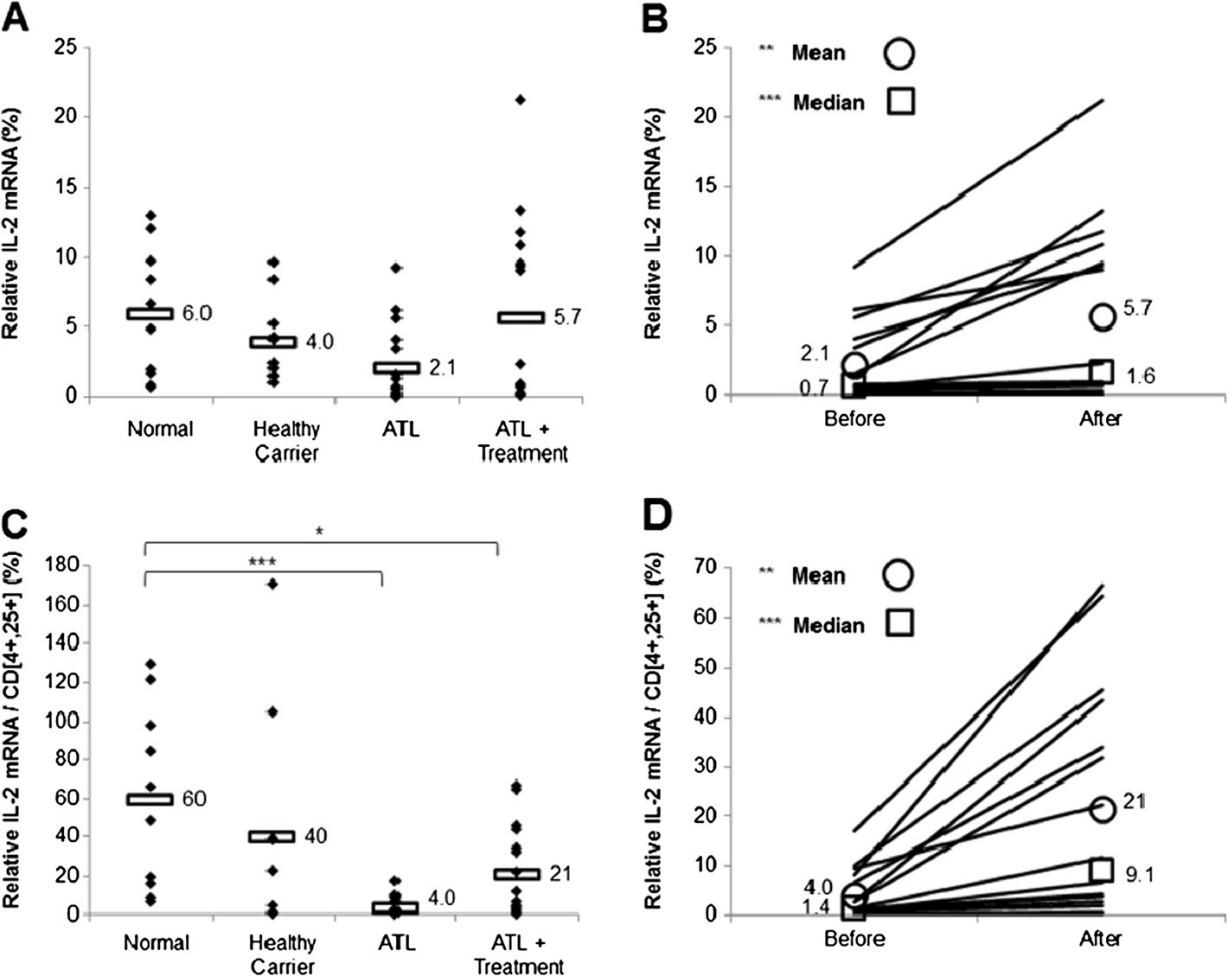
**Treatment with arsenic/IFN/zidovudine increased IL-2 expression. A**. IL-2 transcript levels in normal blood donors (n=10), healthy carriers of HTLV-I (n=10) and ATL patients (n=16) at initiation and 30 days after treatment with arsenic/IFN/zidovudine. Rectangles represent mean values. **B**. Mean and median IL-2 transcript levels of individual ATL patients at initiation and 30 days after treatment with arsenic/IFN/zidovudine. **C**, **D**. Relative expression of IL-2 transcripts after normalization to the number of CD4^+^CD25^+^ ATL cells. All values are expressed as percentage of human beta2-microglobulin used as internal control. *, **, *** indicate p values less than 0.05, 0.01 and 0.001 respectively.

We finally investigated the effect of arsenic/IFN/zidovudine treatment on the Th1 subpopulation by evaluating the transcript levels of IFN-γ and IL-2. ATL patients exhibited significantly lower mRNA levels of IFN-γ (Figure 4A) and normalized IFN-γ (Figure 4C), relative to seronegative or healthy carrier individuals (p < 0.05). Similarly, IL-2 (Figure 5A) and normalized IL-2 (Figure 5C) mRNA levels were lower in ATL patients, compared to seronegative individuals, with only normalized values being statistically significant (p < 0.001). Interestingly, treatment with arsenic/IFN/zidovudine significantly increased the expression and normalized transcript levels of IFN-γ (Table 3 and Figure 4B and D; p < 0.001) and IL-2 (Table 3 and Figure 5B and D; p < 0.01).

## Discussion

In this study, we report an early shift of the cytokine gene expression profile from a T_reg_ and Th2 phenotypes towards a Th1 phenotype in ATL patients after treatment with the combination of arsenic/IFN/zidovudine. We first confirmed the previously reported high transcript levels of Foxp3, IL-10 [12,31,32], and, to lesser extent, IL-4, and low transcript levels of IFN-γ and IL-2, in untreated ATL patients as compared to HTLV-I healthy carriers and HTLV-I negative blood donors. Hence, untreated ATL patients display an immuno-compromised micro-environment characterized by a disruption of the normal cytokine balance, which is skewed toward T_reg_ and Th2 responses. This micro-environment helps ATL cells to evade the immune surveillance and likely explains the frequent opportunistic infections encountered in ATL patients.

The mechanisms of this disruption of the immune system in ATL patients have been extensively investigated. Previous reports indicated that ATL cells functionally and phenotypically resemble T_reg_ cells [13-17]. Indeed, two thirds of ATL cases harbor leukemic cells expressing Foxp3 [33-35]. Furthermore, most ATL cells express T_reg_ markers (CD4, CD25, and Foxp3) suggesting that ATL originates in natural T_reg_ cells infected with HTLV-1 [15,31]. These T_reg_-like ATL cells do not produce IFN-γ [31,36] contrary to the HTLV-I infected CD4^+^CD25^+^ cells in the neuro-inflammatory HTLV-I Associated Myelopathy/Tropical Spastic Paraparesis (HAM/TSP), which display a non-T_reg_ phenotype with decreased expression of Foxp3 and increased levels of IFN- γ [37-41]. Moreover, ATL patients display elevated IL-10 transcript levels contrary to normal T cells [42] and high IL-10 serum level is an unfavorable prognostic factor among ATL patients [31] as well as in HTLV-I negative Hodgkin and non-Hodgkin lymphomas and chronic lymphocytic leukemia [43]. Interestingly, in our study IL-10 transcript level negatively correlates with response at Day 30 (r = −0.452, P < 0.05, spearman), whereas no significant correlations were observed between the standardized expression levels of the other cytokines and response at Day 30 (P > 0.05, spearman). Hence, IL-10 transcript level can be used as biomarker to predict response to arsenic/IFN/zidovudine. Finally, in ATL, IL-10 has strong immunosuppressive effects since it inhibits the proliferation of normal T cells and the expression of Th1 associated cytokines IFN-γ and IL-2 [44,45]. Thus, excessive production of IL-10 by ATL cells may impair the host’s immune system resulting in an immunosuppressive state.

Several studies have demonstrated the role of Th1 cytokines in the control of tumor growth and HTLV-I expression. IL-2 withdrawal was found to induce HTLV-I expression in ATL cell lines [46]. Also, deficiency in IFN-γ resulted in an enhanced tumorigenesis in HTLV-I *tax* transgenic mice [47]. On the other hand, adenovirus-mediated IFN-γ transfer inhibited the growth of transplanted HTLV-I Tax tumors in mice [48]. IFN-γ is produced by the host Th1 cells or natural killer cells as an immune response to HTLV-I infection [11]. ATL cells are unresponsive *in vitro* to TCR stimulation and suppress the proliferation of stimulated T cells [15]. Yano et al. showed that leukemia cells from some ATL patients suppress the proliferation of autologous CD4^+^ non ATL cells, secrete small amounts of IFN-γ, and suppress IFN-γ production by autologous CD4^+^ non-ATL cells [49].

Importantly, as early as one month after treatment with arsenic/IFN/zidovudine, and before achievement of maximal clinical response, we observed significant decreases in Foxp3, IL-10, and IL-4 transcripts and significant increases in IL-2 and IFN-γ expression. The decrease of Foxp3 mRNA correlated with the decreased percentage of CD4^+^CD25^+^ ATL cells. However, significant decreases in IL-10, and, to a lesser extent, IL-4 transcript levels were observed even after adjustment to the number of CD4^+^CD25^+^ leukemic cells. Previous reports have shown that IL-10 and IL-4 are directly produced by ATL cells under the control of Tax-induced NF-кB and Tax activation [50,51]. Hence, decreased IL-10 and IL-4 transcripts in treated ATL patients is likely secondary to arsenic/IFN induced Tax degradation and reversal of NF-кB activation [26,27,29]. Decreased IL-10 expression may alleviate the pre-existing inhibitory effect on Th1 associated cytokines, IFN-γ and IL-2 [44,45], likely explaining the observed increase in their transcript levels after treatment.

This report has several limitations. First, we have only studied the effect of the triple combination of arsenic, IFN and zidovudine, and therefore the individual contribution of each drug alone cannot be assessed. Second, in the absence of functional immune studies, we have only assessed cytokine expression profile which represents an indirect demonstration of the treatment effect on the immune micro-environment. Finally, there is no direct evidence of the mechanism of action and whether the shift of the cytokine expression profile is secondary to the downregulation of HTLV-I proteins (Tax and/or HBZ), inhibition of *de-novo* infection of T cells by HTLV-I, or an HTLV-I independent mechanism. Indeed, while these therapies are clinically quite effective, their mechanism of action remains controversial. Several reports suggested an antiviral effect of zidovudine and interferon without demonstrating it [52]. Preclinical models demonstrated that the combination of arsenic trioxide and interferon eradicate human ATL cells and cures murine ATL derived from Tax transgenics through Tax degradation by the proteasome [29]. However, this has not been demonstrated in ATL patients. Finally, preliminary reports suggested an HTLV-I independent mechanism involving p53 and inhibition of telomerase activity [53].

## Conclusions

In conclusion the shift of the cytokine expression profile from a T_reg_ and Th2 phenotype before treatment toward a Th1 phenotype one month after treatment with arsenic/IFN/zidovudine, and before maximal clinical response and ATL cell loss, may play an important role in restoring an immune response that can participate in the eradication of ATL cells and the prevention or control of opportunistic infections. Therefore, this potentially curative combination can target ATL cells through 1) Tax degradation and eradication of LIC activity (arsenic and IFN), 2) inhibition *de-novo* infection of T cells by HTLV-I (zidovudine and interferon), and 3) switch of the immune system towards an immuno-competent state (triple combination).

## Methods

### Patients’ description, study design and treatment schedule

This study included 16 ATL patients. They were referred between 2006 and 2009 to the Hematology-Oncology Department of Ghaem and Imam Reza hospitals, Mashhad University of Medical Sciences, Iran. All ATL patients had serologic evidence of HTLV-I infection by Enzyme-Linked Immunosorbent Assay (ELISA). Confirmation of HTLV-I positivity was done by standard Polymerase Chain Reaction (PCR) (Data not shown). According to the Shimoyama classification criteria for ATL [18], 12 patients had chronic ATL, 2 patients had acute ATL, while the 2 remaining patients had ATL lymphoma. The patient's characteristics are shown in Table 1. In addition to ATL patients, this study included 10 HTLV-I healthy carriers and 10 HTLV-I seronegative healthy volunteers prospectively selected from the Mashhad Blood Bank Center.

Treatment consisted of intravenous arsenic (10 mg/day, 5 days/week), subcutaneous IFN (Pooyesh Darou Pharmaceutical Co) (5 million units/day), and oral zidovudine (900 mg/day in 3 divided doses) and was administered to the patients for 30 days. Later on, patients received maintenance therapy with zidovudine and IFN. In case of toxicity, zidovudine and IFN were either transiently interrupted or their dose was reduced to 600 mg/day and 3 million units per day, respectively. Arsenic dose was not reduced in case of toxicity, but arsenic treatment was transiently interrupted.

This study was approved by the ethical committee of Mashhad University of Medical Sciences. Blood collection was performed on all patients and control subjects after signing informed consent forms in accordance with the Declaration of Helsinki. Peripheral blood mononuclear cells (PBMCs) and serum were obtained from all the patients before and after 30 days of treatment.

### Response criteria

Complete remission (CR) was defined as a normalization of the CBC associated with a disappearance of all measurable tumors lasting at least one month. Patients with persistence of less than 5% atypical lymphocytes were also considered in CR as this situation may be seen in healthy carriers of HTLV-I. Very good partial response (VGPR) was defined as a normalization of the CBC associated with a disappearance of all measurable tumors lasting at least one month, but with persistence of more than 5% atypical lymphocytes on peripheral blood smear. Partial response (PR) was defined as a decrease of more than 50% in the number of leukemia cells and in the size of all measurable tumors. No response (NR) was defined as less than 50% decrease in the number of leukemia cells or in the size of any measurable tumor, or as disease progression.

### Flow cytometry analysis

Red blood cells were removed using a lysis solution (Becton Dickinson, San Diego). After fixation of white blood cells for 20 minutes in 0.5% paraformaldehyde solution, the cells were washed twice with PBS then immunostained with specific monoclonal antibodies and incubated in dark for 20 minutes. Monoclonal antibodies used for this study were: anti-CD4- peridinin chlorphyl protein (PerCP) , anti-CD3- phycoerythrin (R-PE), anti-CD25-fluorescein isothiocyanate (FITC) and anti-CD8-PE. All monoclonal antibodies (mAbs) were purchased from (IQ-product/Netherlands) and used at 1 in 10 dilution (10 μl of antibody in 100 μl of blood). Flow cytometry data was analysed using the CellQuest software (Becton Dickinson, San Diego). Results are presented as the relative fluorescence intensity and percentage of gated cell populations.

### Quantification of Foxp3 and cytokine expression by real time RT-PCR

Total RNA was isolated from PBMCs using a TriPure Isolation Reagent (Roche Applied Science, Germany). cDNA was synthesized using a RevertAidTM H minus First Strand cDNA Synthesis Kit (Fermentas, Germany) and stored at −20°C until use. Primers were designed at exon-exon junctions (Beacon Designer http://WWW.premierbiosoft.com). All of the selected primers sequences were further analyzed with the Oligo software (http://WWW.cambio.co.uk/index.php). Beta 2 microglobulin (β2m) was used as internal housekeeping control gene to normalize the mRNA expression levels.

Quantification of Foxp3 and cytokine transcript expression was performed using real-time reverse transcriptase (RT) PCR in a rotor gene 6000, Corbett. Taqman method amplification was carried out for Foxp3 using the following primers and probes: Foxp3 sense: 5′ACTACTTCAAgTTCCACAACATgC-3′; Foxp3 anti sense: 5′gAgTgTCCgCTgCTTCTCTg-3′; Foxp3 probe: 5′TCACCTACgCCACgTTCATCCgCT3′; β2m sense: 5′CTTgTCTTTCAgCAAggACTgg-3′; β2m antisense: 5′CCACTTAACTATCTTgggCTgTg-3′; β2m probe: 5′TCACATggTTCACACggCAggCAT-3′. Thermal cycling conditions consisted of an initial step of 10 min at 95°C, followed by 40 cycles at 95°C for 15 s and 60°C for 1 min. Sybergreen amplification was carried out for interleukin-2 (IL-2), IL-4, IL-10, and IFN-γ using the following primers: IL-2 sense CTCACCAggATgCTCACATTTAAg; IL-2 antisense CTCCAgAggTTTgAgTTCTTCTTC; IL-4 sense: CACCgAgTTgACCgTAACAgAC; IL-4 antisense: CCCAggCAgCgAgTgTCC; IL-10 sense: TTgCTggAggACTTTAAgggTTAC; IL-10 antisense: CTTgATgTCTgggTCTTggTTCTC; IFN-γ sense: TgggTTCTCTTgg CTgTTACTg; IFN-γ antisense: gAgTTCCATTATCCgCTACATCTg; β2m sense: CTTgTCTTTCAgCAAggACTgg; β2m antisense: CCACTTAACTATCTTgggCTgTg. Sybergreen reactions were carried out in a final reaction volume of 20 μL using SYBR Premix EX Taq (RR041Q, TaKaRa). Thermal cycling conditions consisted of an initial step of 10 min at 95°C, followed by 40 cycles at 95°C for 10s, 60°C for 30s. For each run, a standard curve was generated using using a five-fold dilution series of a pooled cDNA for Foxp3, IFN-γ, IL-10, IL-2, IL-4 and β2m. The relative standard curves for both reference and target genes were generated by plotting the threshold cycle value versus the log of the dilution of the cDNA. PCR efficiencies (10^-1^/slope^-1^) was automatically calculated by the Rotor Gene Q system Software.

### Proviral load

The HTLV-I viral copy number per μl of blood was calculated from the cell count and the average viral copy number per cell as assessed by quantitative PCR. Real-time quantitative PCR was performed on DNA extracted from peripheral blood mononuclear cells as previously described, using primers and Taqman probe positioned on *tax* gene and *albumin* gene for normalization [30]. TaqMan amplification was carried out in reaction volumes of 25 μL, with the use of the qPCR MasterMix (Eurogentec, Leuven, Belgium). Each sample was analyzed in triplicate with the use of 250 ng of DNA in each reaction. Thermal cycling was initiated with a 2-minutes incubation at 50°C, followed by a first denaturation step of 10 minutes at 95°C and then by 45 cycles at 95°C for 15 seconds and 58°C for 1 minute for *tax* (or 60°C for 1 minute for *albumin*).

### Quantification of IL-10 serum levels

Peripheral blood samples were transferred to serum-separating tubes and centrifuged at 1000g for 20 min after clot formation. The supernatants were carefully harvested, and immediately stored at −80°C until analysis. The serum IL-10 concentration was measured in duplicate by enzyme-linked immunosorbent assay (ELISA) according to the manufacturer’s instructions (R&D Systems, MN, USA). Absorbance of each well was measured spectrophotometrically at 450 nm. The amount of IL-10 protein in the samples was calculated using a reference plot established from serial dilutions of rh IL-10 protein as provided.

### Statistical analysis

SPSS Version 16.0 and Microsoft Office Excel 2010 were used for statistical analyses. In scatter plots, means were compared using ANOVA with associated *post-hoc* tests: Dunnett *t*, Tukey, Student-Newman-Keuls (SNK), and Bonferroni tests. All analyzed samples fit normal distributions, except IFN-γ and IL-10 values in ATL patients. Therefore, both mean and median were plotted for ATL patient data, as shown in line diagrams, and were analyzed using parametric and nonparametric (Wilcoxon) paired sample *t*-tests, respectively, to compare values before and after treatment. In all cases, mean and median either both showed statistical significance or both not. For normally distributed data, pearson correlation was performed. Otherwise, spearman correlation was used and compared to pearson’s rho tested on log-transformed data, with similar results. Spearman rho was employed for correlations involving ordinal scale data. Statistical significance was reported when the *P-value* was ≤ 0.05, except in scatter plots comparing ATL patients before and after treatment, for which *P-values* were stated separately in line diagrams. *, P < 0.05; **, P < 0.01; ***, P < 0.001.

## Competing interests

The authors declare that they have no competing interests.

## Authors' contributions

GK and MT performed diagnostic and molecular analysis and patients’ follow up, participated in study design, data analysis and write up of the manuscript. SAR R, RF, H Rafatpana, AG and MM participated in study design, data analysis and write up of the manuscript. AG and HE performed statistical analysis and participated in data analysis and write up of the manuscript. MK, H Rahimi and AS treated patients and participated in data analysis. FB, MM, RN, ZD and ME participated in data analysis. HD and OH participated in study design, data analysis and write up of the manuscript. AB designed the study and wrote the manuscript. All authors read and approved the final manuscript.
